# Role of Unfolded Protein Response and Endoplasmic Reticulum-Associated Degradation by Repeated Exposure to Inhalation Anesthetics in *Caenorhabditis elegans*

**DOI:** 10.7150/ijms.58043

**Published:** 2021-06-01

**Authors:** Saeyeon Kim, Hyun-Jung Shin, Sang-Hwan Do, Hyo-Seok Na

**Affiliations:** Department of Anesthesiology and Pain Medicine, Seoul National University Bundang Hospital, Seongnam, South Korea.

**Keywords:** *Caenorhabditis elegans*, endoplasmic reticulum-associated degradation pathway, inhalation anesthetics, unfolded protein response

## Abstract

**Background:** When an imbalance occurs between the demand and capacity for protein folding, unfolded proteins accumulate in the endoplasmic reticulum (ER) lumen and activate the unfolded protein response (UPR). In addition, unfolded proteins are cleared from the ER lumen for ubiquitination and subsequent cytosolic proteasomal degradation, which is termed as the ER-associated degradation (ERAD) pathway. This study focused on changes in the UPR and ERAD pathways induced by the repeated inhalation anesthetic exposure in *Caenorhabditis elegans*.

**Methods**: Depending on repeated isoflurane exposure, *C. elegans* was classified into the control or isoflurane group. To evaluate the expression of a specific gene, RNA was extracted from adult worms in each group and real-time polymerase chain reaction was performed. Ubiquitinated protein levels were measured using western blotting, and behavioral changes were evaluated by chemotaxis assay using various mutant strains.

**Results**: Isoflurane upregulated the expression of *ire-1* and *pek-1* whereas the expression of *atf-6* was unaffected. The expression of both *sel-1* and *sel-11* was decreased by isoflurane exposure, possibly indicating the inhibition of retro-translocation. The expression of *cdc-48.1* and *cdc-48.2* was decreased and higher ubiquitinated protein levels were observed in the isoflurane group than in the control, suggesting that deubiquitination and degradation of misfolded proteins were interrupted. The chemotaxis indices of *ire-1*, *pek-1*, *sel-1*, and *sel-11* mutants decreased significantly compared to N2, and they were not suppressed further even after the repeated isoflurane exposure.

**Conclusion**: Repeated isoflurane exposure caused significant ER stress in *C. elegans*. Following the increase in UPR, the ERAD pathway was disrupted by repeated isoflurane exposure and ubiquitinated proteins was accumulated subsequently. UPR and ERAD pathways are potential modifiable neuroprotection targets against anesthesia-induced neurotoxicity.

## Introduction

General anesthesia is an essential practice for various surgeries, and inhalation anesthetics are commonly used for general anesthesia, either alone or in combination with other drugs. Over the past decade, extensive pre-clinical researches have consistently shown that anesthetic exposure during early post-natal period can cause the neurotoxicity leading to the behavioral or cognitive defects 1-3. However, it is still controversial whether the animal studies can translate to clinical field. Recent three studies 4-6 including large population stated that developmental neurotoxicity may not exist in single brief anesthetic exposure during early life. In 2016, the U.S. Food and Drug Administration issued a warning regarding anesthetic and sedative agents describing the potential risk of anesthesia-induced neurotoxicity (AIN) in children aged below 3 years, particularly when they are exposed to these drugs over a long term or repeatedly 7.

Diverse mechanisms related to cell death, growth factor signaling, mitochondria, N-methyl D-aspartate, γ-aminobutyric acid, or the endoplasmic reticulum (ER) have been postulated as an underlying process of AIN 8. In addition, the mammalian target of rapamycin signaling pathway 9 and brain-derived neurotrophic factor were known to be modulated by anesthesia in developing nervous system 10. However, the precise mechanism underlying AIN has not yet been determined, and this study focused on ER stress and the following changes in the unfolded protein response (UPR) responses and ER-associated degradation (ERAD) pathway after repeated inhalation anesthetic exposure.

The ER is an intracellular organelle that facilitates the folding and maturation of protein molecules and their transport to the Golgi apparatus. The release of excessive Ca^2+^ from the ER and the relationship of ryanodine receptors have been studied as a causative factor of anesthesia-induced ER stress 11-13. Excessive cytosolic Ca^2+^ changes the protein-folding environment in the ER, leading to ER stress 14. When an imbalance occurs between the demand and capacity for protein folding, unfolded or misfolded proteins accumulate in the ER lumen and activate UPR 15. In addition, misfolded proteins are cleared from the ER lumen for ubiquitination and subsequent cytosolic proteasomal degradation, which is termed as ERAD pathway 16. This process controls the quality and quantity of proteins.

*Caenorhabditis elegans* contains several orthologous genes involved in the human UPR and ERAD pathways, and they were evaluated in several conditions such as aging or neurodegenerative diseases 17, 18. This study evaluated changes in the UPR and ERAD pathways induced by the repeated inhalation anesthetic exposure in *Caenorhabditis elegans*.

## Methods

### Caenorhabditis elegans strains and anesthesia exposure

Wild-type N2, *zcls4[hsp-4::GFP]V*, *ire-1(ok799) II*, *pek-1(ok275) X*, *atf-6(ok551) X*, *sel-1(mg547) V*, *cdc-48.1(tm544) II*, and *cdc-48.2(tm659) II C. elegans* were obtained from the Caenorhabditis Genetics Center (Minneapolis, MN, USA). RNA interference (RNAi) was performed when the *sel-11* strain was used. Culturing, synchronization, anesthesia, and chemotaxis assay were performed according to the methods described in our previous paper 19. Isoflurane was used for the anesthesia of *C. elegans*, and was administered four times during each stage from L1 to L4. The concentration of isoflurane was the 99.9% effective immobilizing dose, which was determined by pilot examination and used in our previous study 19. Depending on isoflurane exposure, the worms were classified into the control or isoflurane group.

### RNA preparation and real-time polymerase chain reaction (PCR)

To evaluate the expression of a specific gene, RNA was extracted from adult worms in each group. After collecting the worm pellet, it was ground to a fine frozen powder using liquid nitrogen. After adding a RLT butter, ethanol, and RW1 buffer sequentially to the frozen powdered worm, purified RNA was extracted using RNeasy mini spin column. Finally, RNase-free water was added to the extracted RNA, which was frozen at -80°C until use. Using a NanoDrop (ND-2000, Thermo Fisher Scientific, MA, USA) and an Agilent 2100 Bioanalyzer (Agilent Technologies, Palo Alto, USA), the ratios of absorbance at 260-280 nm (OD 260/280) and at 260-230 nm (OD 260/230) were determined and the quality-controlled RNA (OD 260/280 >1.5 and OD 260/230 >1.0) was used for real-time polymerase chain reaction. After first-strand cDNA synthesis using Maxima H minus First strand cDNA Synthesis Kit (Thermo Fisher Scientific, MA, USA), real-time PCR was performed using the cDNA, each gene-specific primer (Table 1), and Power SYBT Green PCR Master mix (Applied Biosystems, MA, USA). Pan-actin was used as a reference gene and the ΔCT was calculated.

### Western blotting

*C. elegans* were washed with S-basal buffer three times and lysed in cold RIPA lysis buffer (BRA0500, BIOMAX; 25 mM Tris-HCl, pH 7.6, 150 mM NaCl, 1% NP-40, 1% sodium deoxycholate, 1% sodium dodecyl sulfate) containing 100 mM iodoacetamide. Protein concentrations were measured using a Pierce BCA Protein Assay Kit (Thermo Fisher Scientific, MA, USA). Equal amounts of target proteins, normalized to the actin level, in 2X Laemmli sample buffer (Bio-Rad, CA, USA) were heated at 95 °C for 5 min. Proteins were resolved by 8-15% sodium dodecyl sulfate-polyacrylamide gel electrophoresis and transferred to polyvinylidene difluoride membranes. After blocking with 5% skim milk in a Tris-buttered saline with0.1% Tween® 20 buffer (20 mM Tris, 500 mM sodium chloride, and 0.1% Tween 20, pH 7.5) for 1 h at 20 °C, the membranes were incubated overnight at 4 °C with anti-ubiquitin antibody (1:1000; Abcam, Cambridge, UK) and anti-actin antibody (1:5000; Abcam, Cambridge, UK). After secondary antibody treatment for 1 h at 20 °C, the membranes were developed using an enhanced chemiluminescence kit (DG-W250, DoGen, Korea). Horseradish peroxidase-conjugated secondary antibodies (sc-516102) were obtained from Santa Cruz Biotechnology (Texas, USA).

### Fluorescence imaging

In *zcls4[hsp-4::GFP]V* strain, green fluorescence protein (GFP) expression was measured using a Zeiss LSM 710 confocal microscope system (Oberkochen, Germany). L4 stage worms were mounted on agar pad after immobilizing them by sodium azide.

### Chemotaxis assay

**Figure 1:**
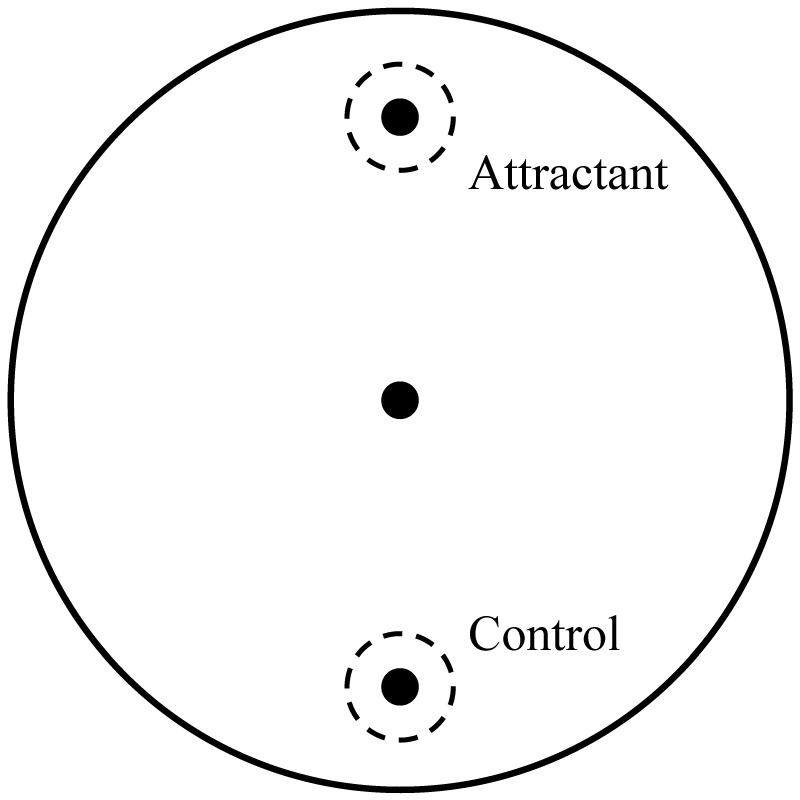
** Chemotaxis assay plate.** A 9-cm petri plate covered with nutrient growth medium was used for chemotaxis assay. OP50 was used for attractant, and control site was blank. Before placing worm pellet in the center, 1 μl of 1 M sodium azide was dropped in both sites to immobilize worms when they reached there. Number of worms in a circle within 1.5-cm radius at each point and in other zone was counted and used for chemotaxis index calculation.

Chemotaxis assay was performed by one experimenter blind to condition to confirm the abnormal behavioral pattern when *C. elegans* reached the young adult stage as described in our previous study 19. About 50 young adult worms, which were washed by S-basal buffer, were transferred to the center of the chemotaxis plates (Fig. 1). After 1 h, the number of the worms on each side was counted and chemotaxis index was calculated using the equation: (number of A point - number of C point)/total number of worms 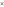 100 (%). Chemotaxis assay was performed three times and all batches included 3 plates in each group.

### Statistics

Data are presented as the mean and standard deviation. Due to the small sample size, nonparametric test, Mann-Whitney U test, was used to determine the significance of differences between the two groups. Statistical analyses were performed using SPSS (version 21.0; IBM Co., Armonk, NY, USA), and P values of < 0.05 were considered to indicate significant differences.

## Results

HSP-4, a heat shock protein in *C. elegans*, is used to monitor ER stress and it is homologous to binding immunoglobulin protein (BiP) in mammals. The ER-specific heat shock protein HSP-4 reporter (*Phsp-4::gfp*) was upregulated in L1 larvae after isoflurane exposure, as reported previously 20. After repeated isoflurane exposure during the developmental period, the expression of *hsp-4::GFP* was increased (Fig. S1). Real-time PCR was performed to validate the expression of *hsp-4*. In isoflurane-exposed worms, the expression of *hsp-4* was induced (1.4 ± 0.1 in isoflurane group compared to the normalized control group; P <0.001).

**Figure 2:**
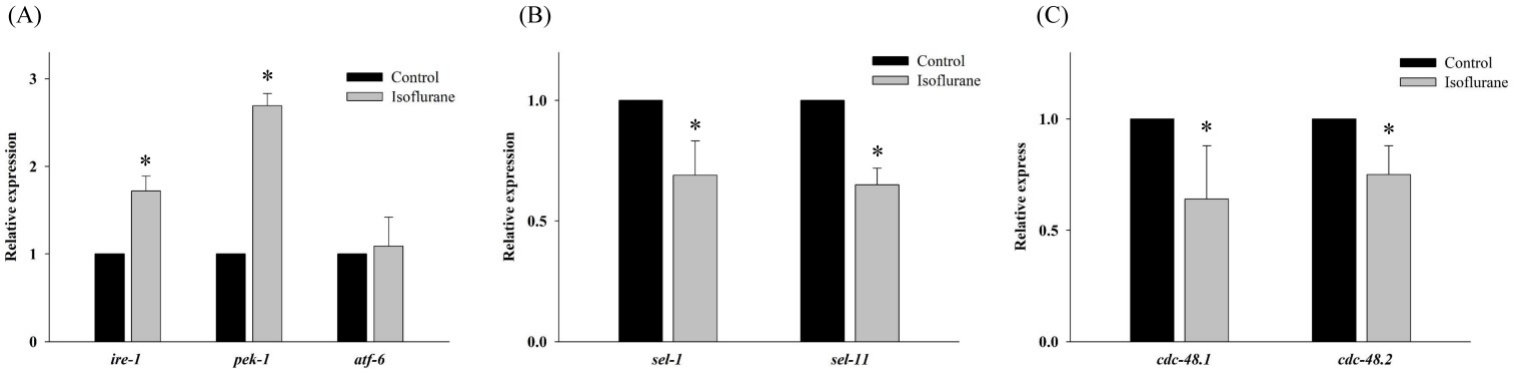
** Expression of genes related to the unfolded protein response and endoplasmic reticulum associated degradation.** (A) Isoflurane upregulated the expression of *ire-1* and *pek-1*; however, the expression of *atf-6* remained unaffected. (B) The expression of both *sel-1* and *sel-11* was decreased by isoflurane exposure. (C) The expression of *cdc-48.1* and *cdc-48.2* was decreased by isoflurane exposure. All batches included 3 plates in each group and same assay was performed three times. Error bar, standard deviation; *P<0.001.

To determine the effects of isoflurane on the regulation of the UPR and ERAD pathway in AIN, the expression of related genes was evaluated by real-time PCR under the same conditions. Further, *ire-1*, *pek-1*, and *atf-6* correspond to inositol-requiring enzyme 1 (IRE1), protein kinase RNA-like ER kinase (PERK), and activating transcription factor 6 (ATF6) in humans and are related to UPR in *C. elegans*. Isoflurane upregulated the expression of *ire-1* and *pek-1* (P <0.001 in both); however, the expression of *atf-6* (P = 0.726) remained unaffected (Fig. 2A).

**Figure 3:**
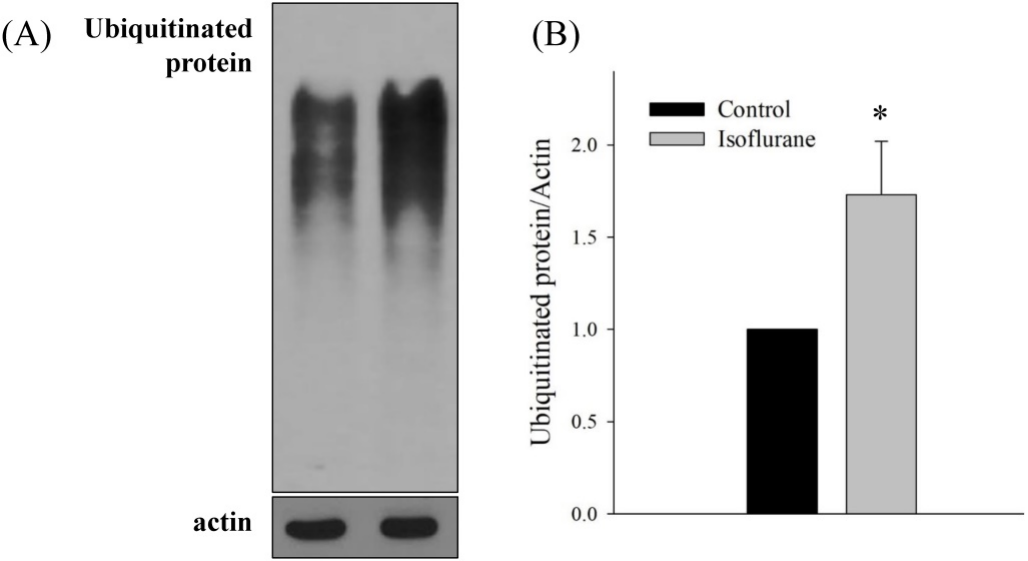
** Western blotting for ubiquitinated proteins.** (A, B) Higher levels of ubiquitinated protein were observed in the isoflurane group. All batches included 3 plates in each group and same assay was performed three times. Error bar, standard deviation; *P<0.001.

Both *sel-1* and *sel-11* are orthologs of human SEL-1L and HRD1, which are related to the ERAD pathway and induced by ER stress. Interestingly, the expression of both *sel-1* and *sel-11* was decreased by isoflurane exposure (P <0.001 in both) (Fig. 2B). Polyubiquitin chains bind to proteins destined for degradation to serve as a degradation signal. Both *cdc-48.1* and *cdc-48.2* are orthologs of P97, which facilitates the degradation of large amounts of misfolded proteins. The expression of *cdc-48.1* and *cdc-48.2* was decreased by isoflurane exposure (P <0.001 in both) (Fig. 2C). The decreased expression of *sel-1*, *sel-11*, *cdc-48.1* and *cdc-48.2* suggests that the ERAD pathway was inhibited. Therefore, the levels of ubiquitinated proteins were investigated by western blotting with an anti-ubiquitin antibody. Higher levels of ubiquitinated protein were observed in the isoflurane group than in the control group (P <0.001), suggesting that ERAD was interrupted by isoflurane (Fig. 3).

**Figure 4:**
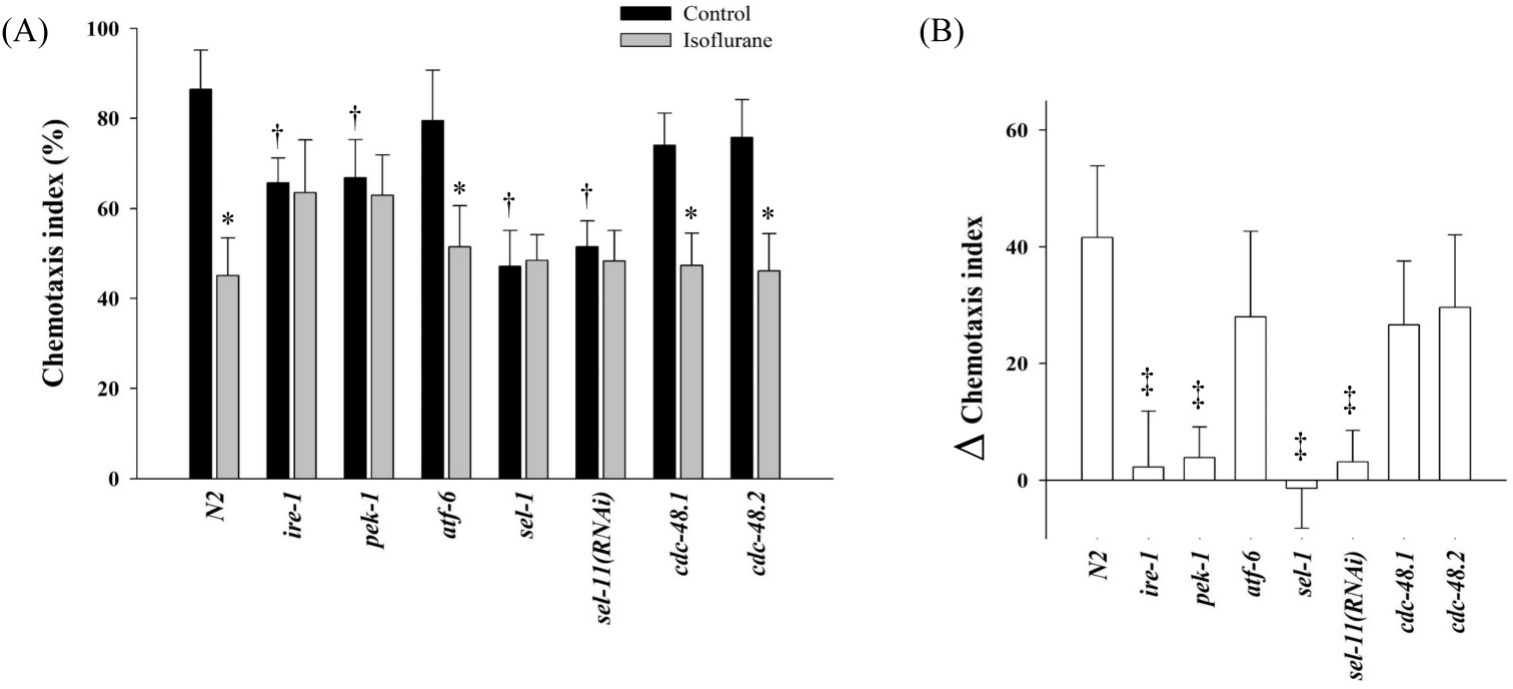
** Chemotaxis index.** In several mutant strains related unfolded protein response and endoplasmic reticulum-associated degradation, the chemotaxis indices were measured (A) and the difference between the control and isoflurane group was calculated in each strains (B). All batches included 3 plates in each group and same assay was performed three times. Error bar, standard deviation; *P <0.05 vs. control in each strain; †P = 0.001 vs. N2 control group; ‡P <0.001 vs. N2.

The chemotaxis index of N2 was 86.4 ± 8.8% in the control group, whereas it was 45.1 ± 8.4% in the isoflurane group (P = 0.001). In several mutant strains related UPR and ERAD, the chemotaxis indices were measured (Fig. 4). In the control groups, the chemotaxis indices of *ire-1*, *pek-1*, *sel-1*, and *sel-11(RNAi)* decreased significantly compared to those of N2 (P < 0.001). The chemotaxis indices were not suppressed further even after the repeated isoflurane exposure in these four mutants.

## Discussion

We found that repeated exposure to isoflurane induced the expression of ER chaperones and UPR in *C. elegans*, indicating an increase of ER stress. To the best our knowledge, it is observed for the first time that ubiquitinated proteins were accumulated considerably with a disrupted ERAD pathway in the isoflurane group than in the control group.

BiP is an ER chaperone present on the ER membrane in the absence of ER stress. However, it dissociates from the ER membrane and binds to misfolded proteins during ER stress 21. In addition, three membrane proteins, namely PERK, ATF6, and IRE1, are activated and initiate the UPR signaling pathway 22.

In *C. elegans*, *hsp-4*, *pek-1*, *atf-6*, and *ire-1* are the homologues of human BiP, PERK, ATF6, and IRE1, respectively. It is known that *hsp-4* is induced under ER stress in *C. elegans* and their UPR is very similar to that of humans 23. Higher expression of *hsp-4* after repeated exposure to isoflurane indicates that isoflurane induces ER stress in *C. elegans*; a similar result was observed in a previous study 20. Interestingly, two major UPR regulators, *pek-1* and *ire-1*, were induced in isoflurane-exposed *C. elegans,* whereas *atf-6* was not affected. We could not determine the exact cause of the gene expression mismatch among the three UPR regulators; however, the activation processes differed among the three UPR genes. A previous study reported that IRE1 and PERK have a similar luminal domain and detect unfolded proteins through the same mechanism 24. IRE1 and PERK exist in an inactive state by binding with BiP in the absence of ER stress. However, ER stress causes BiP to dissociate from the luminal domains of IRE1 and PERK, resulting in their oligomerization and autophosphorylation 25. In contrast, ER stress causes the translocation of ATF6 from the ER to the Golgi, where it is cleaved to its active form 26 that becomes dominant in the absence of ER stress 27. Thus, cleaved *atf-6* might not be detected by real-time PCR, which caused the discrepancy in gene expression of the three UPR regulators in this study.

Misfolded proteins are retrotranslocated from the ER lumen into the cytosol, and they are recognized and cleared by the ERAD pathway to maintain ER homeostasis 28. HRD1 and SEL-1L, the most representative ERAD complex, correspond to *sel-11* and *sel-1* in *C. elegans*, respectively. Previously, deletion of HRD1 or SEL1L in mice was reported to cause embryonic or premature death 29-31. The pathobiological role of the E3 ubiquitin ligase HRD1 and its adaptor protein SEL1L was previously evaluated, and their importance was demonstrated in neurodegenerative diseases. Both were proposed to be involved in the pathogenesis of and identified as therapeutic targets against Parkinson's disease 32, 33. HRD1 was also involved in the accumulation of the amyloid precursor protein and the subsequent production of amyloid β, which is linked to Alzheimer's disease 34-36. Polymorphism in SEL1L may also be a susceptibility factor for Alzheimer's disease 37. According to our results, the expression of *sel-11* and *sel-1* was suppressed by repeated isoflurane exposure, which might have increased the *hsp-4::GFP* expression sequestrated to the misfolded protein in the ER lumen.

Retrotranslocated misfolded proteins are modified with ubiquitin, and p97 (also known as valosin-containing protein) guides these proteins to the proteasome for degradation 38. P97 is a component of the ERAD pathway and its gene deletion can have fatal consequences 39. The orthologs of p97 are *cdc-48.1* and *cdc-48.2* in *C. elegans*, and repeated isoflurane-associated decreases in these genes induced aggregation of polyubiquitin-conjugated proteins. The defective ubiquitin-proteasome system interrupts the degradation of retrotranslocated misfolded proteins via the proteasome. Accumulation and aggregation of neurotoxic proteins by dysregulation of the ubiquitin-proteasome system is known to be associated with numerous neurodegenerative diseases 40. Particularly, polyglutamine aggregation causes several neurodegenerative diseases including Huntington's or Machado-Joseph disease, and p97 homologs have been reported to play a protective role in polyglutamine aggregates 18, 41. Although we could not identify whether the accumulated ubiquitinated proteins act as toxic aggregates, we found that repeated isoflurane exposure might interrupt the elimination of aggregate formation in *C. elegans*. A previous study showed that inhalation anesthetic can induce neuronal protein aggregation and mislocalization 42.

Behavioral change was evaluated by chemotaxis assay in our study. The significant decrease in chemotaxis index after repeated isoflurane exposure in wild-type N2 might be caused by increased ER stress and defective ERAD. UPR and ERAD pathway essentially play a role in the stress response, and the aforementioned results were observed in the nervous system as well as in the non-neuronal tissue throughout the entire body of *C. elegans*; however, they are finally involved in maintaining a variety of physiological conditions in a normal state 17, 23, 43. Thus, it might be said that developmental repeated exposure of *C. elegans* to isoflurane worsen the chemotaxis index.

Interestingly, several mutants showed different patterns in chemotaxis indices. Loss of *ire-1* or *pek-1* was known to affect various basal physiology, such as secretory-protein metabolism, longevity, or development 43-45; therefore, the basal chemotaxis indices of* ire-1* and *pek-1* mutants seemed to decrease in the control group compared to that of N2. Unlike *ire-1* or *pek-1*, the *atf-6* mutants were known to have extended lifespan without sensitivity to proteotoxic stress 46, 47, and the results of chemotaxis index were not different from that of N2. The other two mutants, *sel-1*and *sel-11*, also showed different chemotaxis indices from those of N2. Loss of *sel-1* or *sel-11* is able to induce ER stress 48 or lead to behavioral defects 49, respectively, which appeared to decrease chemotaxis index in each control group of *sel-1* and *sel-11* compared to the N2 control. Moreover, repeated isoflurane no longer suppressed the chemotaxis indices further in *ire-1*, *pek-1, sel-1,* and *sel-11*, which could be interpreted that those four genes might be significantly affected by repeated isoflurane exposure during developmental period in *C. elegans*. Both *cdc-48.1* and *cdc-48.2* were known to act redundantly in the elimination of misfolded protein from ER 50, 51; thus, either *cdc-48.1* or *cdc-48.2* single mutant did not seem to present any difference from the N2 in their chemotaxis assay.

This study had several limitations. First, it is unclear if suppression of each gene may result in decreased levels of each protein. We could not investigate all protein levels involved in the UPR and ERAD pathway because of antibody unavailability for *C. elegans*. Furthermore, we did not investigate the phosphorylation of each UPR gene or subsequent initiation of downstream signaling events. Finally, this study was conducted in *C. elegans*. Although this model is valuable for studying certain signaling pathways and cellular processes, which have been well-conserved in humans, it is unclear whether our conclusion can be extrapolated to humans. Despite these limitations, our findings revealed that repeated isoflurane exposure leads to defective expression of genes associated with the UPR and ERAD pathways. Studies are needed to determine how this abnormal gene expression influences potential neurodegenerative consequences by anesthetic agents.

In conclusion, we showed that repeated isoflurane exposure caused significant ER stress in *C. elegans*. Following the increase in UPR, the ERAD pathway was disrupted by repeated isoflurane exposure and ubiquitinated proteins was accumulated subsequently. UPR and ERAD pathways are potential vulnerable neuroprotective targets against anesthesia-induced neurotoxicity.

## Supplementary Material

Supplementary figures and tables.

## Figures and Tables

**Table 1:** Forward and Reverse Primer Sequences for Real-Time PCR

Primer	Sequence
***ire-1***	
Forward	ACAATGGCTAGTCAGCGAGG
Reverse	CTTCTGGAGCAATCCAGCCA
***pek-1***	
Forward	TGACATTGACACCGACGAGG
Reverse	TGCCCGATGACCTTCTTGAC
***atf-6***	
Forward	ATCGTTGCTCCTGCCTAGTG
Reverse	TCAATTGGCCAGTCCCTGTC
***sel-1***	
Forward	GTGGACGAGGGCTCAATCAA
Reverse	AATGCATCGGCACTTCCTGA
***sel-11***	
Forward	GCGTCTTCCACACCAACAAC
Reverse	CCTAGAAGACGTGCTAGGCG
***cdc-48.1***	
Forward	TGCTCACAATGTGGTTCGGA
Reverse	GAACAACACGCAAGGAGCAG
***cdc-48.2***	
Forward	GAGAAGCGTATCGTCTCGCA
Reverse	TTAGTAGCGGCGATCACGAC
***pan-actin***	
Forward	TCGGTATGGGACAGAAGGAC
Reverse	CATCCCAGTTGGTGACGATA
