# Supplementary figures and images for: Role of Unfolded Protein Response and Endoplasmic Reticulum-Associated Degradation by Repeated Exposure to Inhalation Anesthetics in Caenorhabditis elegans
Source: Int J Med Sci. 2021 Jun 1;18(13):2890–6. doi: 10.7150/ijms.58043 (PMC8241789; doi:10.7150/ijms.58043)

**Figure S1. Change of the expression of *hsp-4* after repeated isoflurane exposure.**

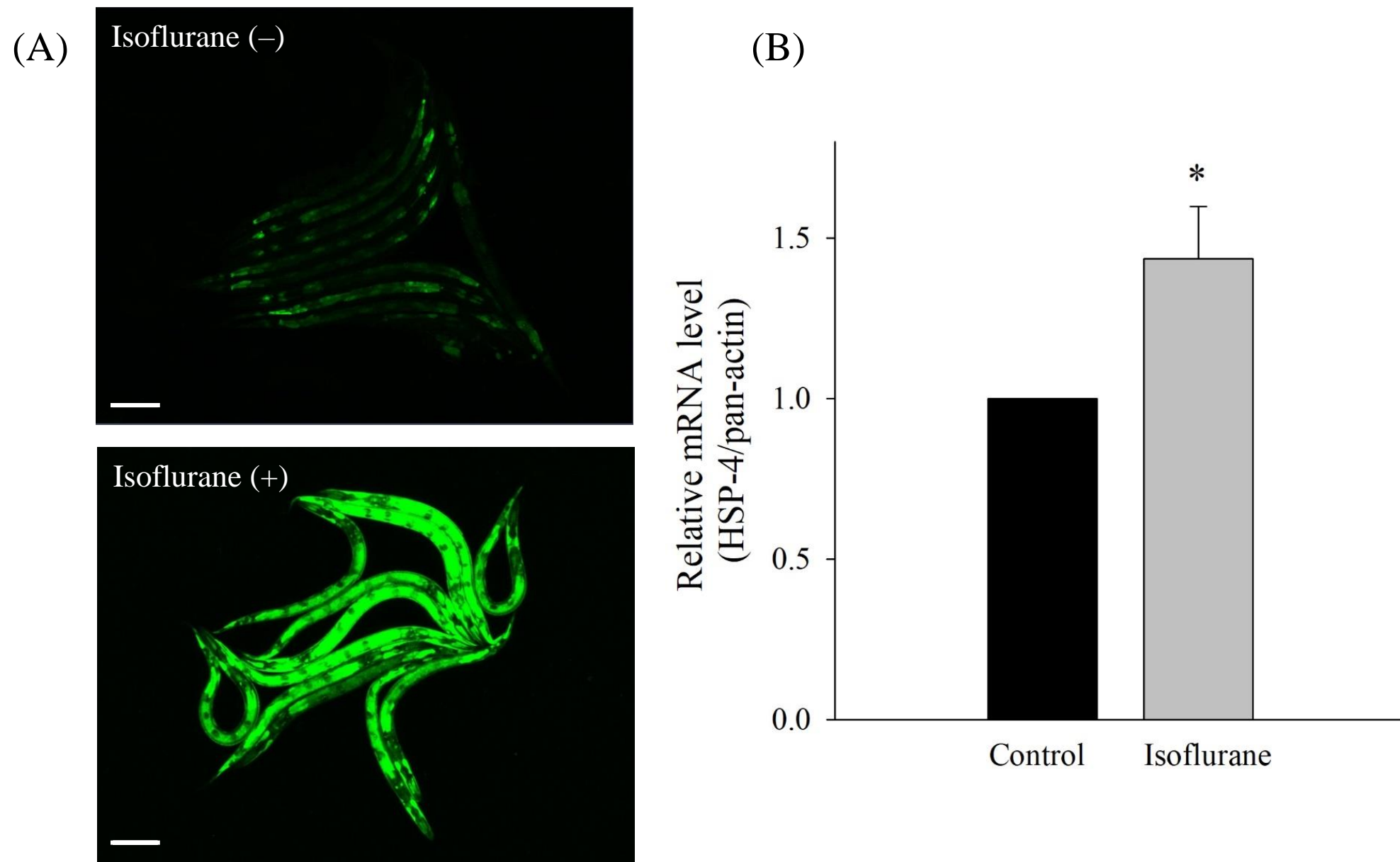

Supplement: Supplementary file 1 — Supplementary figures and tables. [file ijmsv18p2890s1.pdf]
